# Supplementary material for: Splice-Junction-Based Mapping of Alternative Isoforms in the Human Proteome
Source: Cell Rep. Author manuscript; Available in PMC 2020 Jan 15. (PMC6961840; doi:10.1016/j.celrep.2019.11.026)
Supplement: 3 [file NIHMS1546469-supplement-3.zip › DF2/PXD0010154/Lung-301-P02751-KKTGQEALSQTTISWAPF.pdf]

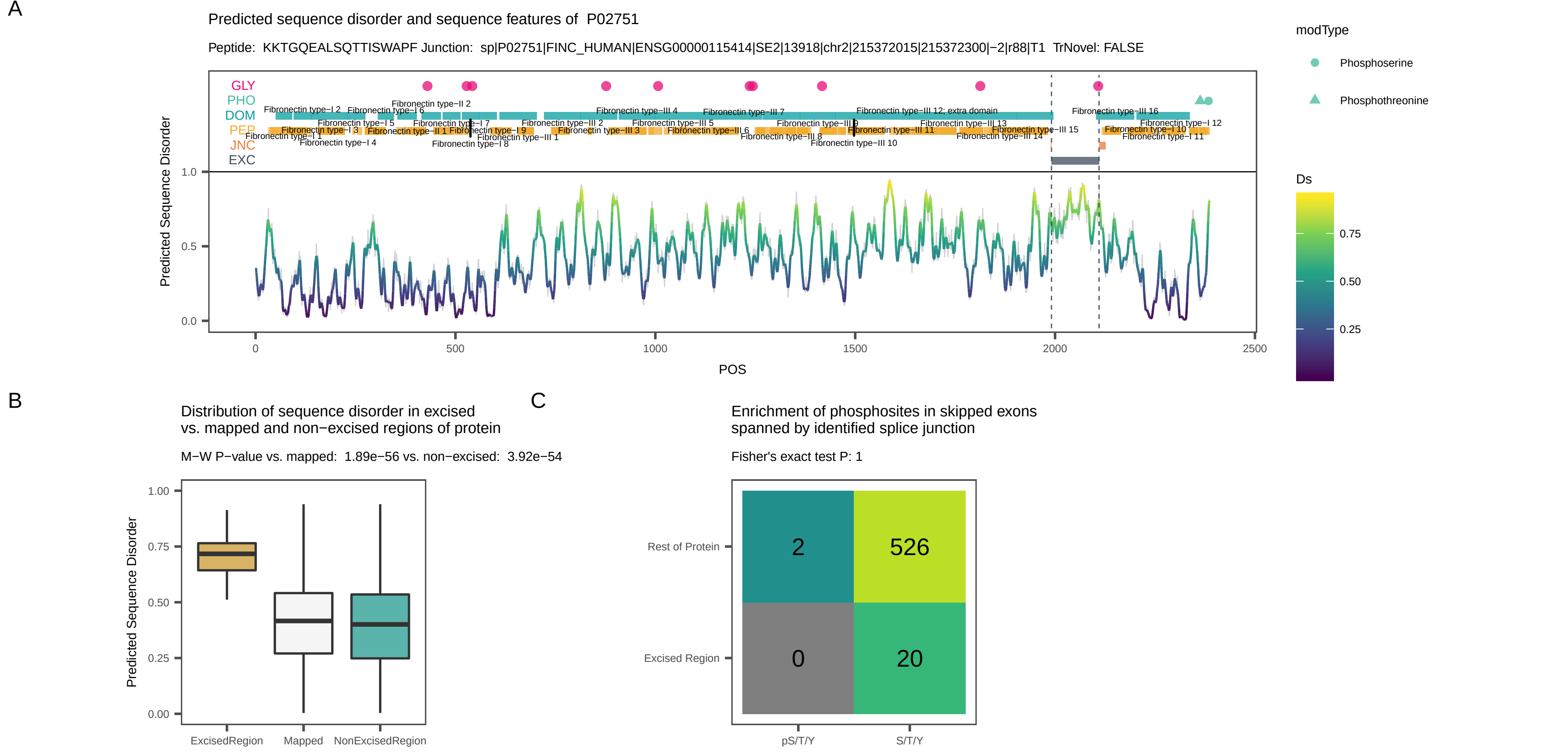
A
Predicted sequence disorder and sequence features of P02751
Peptide: KKTGQEALSQTTISWAPF Junction: sp|P02751|FINC_HUMAN|ENSG00000115414|SE2|13918|chr2|215372015|215372300|−2|r88|T1 TrNovel: FALSE
GLY
PHO
DOM
PEP
JNC
EXC
Fibronectin type−I 2
Fibronectin type−I 6
Fibronectin type−II 2
Fibronectin type−III 4
Fibronectin type−III 7
Fibronectin type−III 12; extra domain
Fibronectin type−III 16
Fibronectin type−I 5
Fibronectin type−I 7
Fibronectin type−III 2
Fibronectin type−III 5
Fibronectin type−III 9
Fibronectin type−III 13
Fibronectin type−I 12
Fibronectin type−I 3
Fibronectin type−II 1
Fibronectin type−I 9
Fibronectin type−III 3
Fibronectin type−III 6
Fibronectin type−III 11
Fibronectin type−III 15
Fibronectin type−I 10
Fibronectin type−I 1
Fibronectin type−III 1
Fibronectin type−III 8
Fibronectin type−III 14
Fibronectin type−I 11
Fibronectin type−I 4
Fibronectin type−I 8
Fibronectin type−III 10
Predicted Sequence Disorder
1.0
0.5
0.0
0
500
1000
1500
2000
2500
POS
modType
Phosphoserine
Phosphothreonine
Ds
0.75
0.50
0.25
B
Distribution of sequence disorder in excised vs. mapped and non−excised regions of protein
M−W P−value vs. mapped: 1.89e−56 vs. non−excised: 3.92e−54
Predicted Sequence Disorder
1.00
0.75
0.50
0.25
0.00
ExcisedRegion
Mapped
NonExcisedRegion
C
Enrichment of phosphosites in skipped exons spanned by identified splice junction
Fisher's exact test P: 1
Rest of Protein
Excised Region
2
526
0
20
pS/T/Y
S/T/Y
